# Enhancing hepatoprotective action: oxyberberine amorphous solid dispersion system targeting TLR4

**DOI:** 10.1038/s41598-024-65190-2

**Published:** 2024-06-28

**Authors:** Tingting Chen, Qingguo Li, Gaoxiang Ai, Ziwei Huang, Jun Liu, Lingfeng Zeng, Ziren Su, Yaoxing Dou

**Affiliations:** 1 Meizhou Hospital of Guangzhou University of Chinese Medicine (Meizhou Hospital of Traditional Chinese Medicine), 3 Huanan Avenue, Meijiang District, Meizhou, Guangdong China; 2 School of Pharmaceutical Sciences, Guangzhou University of Chinese Medicine, Guangzhou, China; 3 The Second Clinical Medical College of Guangzhou University of Chinese Medicine/Post-Doctoral Research Station, Guangzhou, China; 4 The Second Affiliated Hospital of Guangzhou University of Chinese Medicine (Guangdong Provincial Hospital of Chinese Medicine), Guangzhou, China; 5 Guangdong Second Traditional Chinese Medicine Hospital (Guangdong Province Enginering Technology Research Institute of Traditional Chinese Medicine), Guangzhou, China; 6 Bone and Joint Research Team of Degeneration and Injury, Guangdong Provincial Academy of Chinese Medical Sciences, Guangzhou, China; 7 School of Medicine, Southern University of Science and Technology, Shenzhen, China; 8 Institute of Animal Husbandry and Veterinary Science, Jiangxi Academy of Agricultural sciences, Nanchang, China

**Keywords:** Acute liver injury, Oxyberberine, Amorphous solid dispersions, Bioavailability, Pharmaceutics, Pharmacology

## Abstract

Oxyberberine (OBB) is a significant natural compound, with excellent hepatoprotective properties. However, the poor water solubility of OBB hinders its release and absorption thus resulting in low bioavailability. To overcome these drawbacks of OBB, amorphous spray-dried powders (ASDs) of OBB were formulated. The dissolution, characterizations, and pharmacokinetics of OBB-ASDs formulation were investigated, and its hepatoprotective action was disquisitive in the D-GalN/LPS-induced acute liver injury (ALI) mouse model. The characterizations of OBB-ASDs indicated that the crystalline form of OBB active pharmaceutical ingredients (API) was changed into an amorphous form in OBB-ASDs. More importantly, OBB-ASDs showed a higher bioavailability than OBB API. In addition, OBB-ASDs treatment restored abnormal histopathological changes, improved liver functions, and relieved hepatic inflammatory mediators and oxidative stress in ALI mice. The spray drying techniques produced an amorphous form of OBB, which could significantly enhance the bioavailability and exhibit excellent hepatoprotective effects, indicating that the OBB-ASDs can exhibit further potential in hepatoprotective drug delivery systems. Our results provide guidance for improving the bioavailability and pharmacological activities of other compounds, especially insoluble natural compounds. Meanwhile, the successful development of OBB-ASDs could shed new light on the research process of poorly soluble medicine.

## Introduction

Acute liver injury (ALI) is a deadly clinical situation, which is characterized by a rapid loss and abnormality of hepatocyte function^1^. In general, viruses, bacteria, drugs, toxins, and alcohol are the main risk factors of ALI^2^. The pathogenesis of ALI is complicated, and uncontrolled inflammation has been widely considered as the primary underlying mechanism^3^.

8-Oxyberberine (OBB, PubChem CID: 11066; the structure is shown in Table 1) is a significant natural compound that was isolated from a variety of plants^4^. The current research shows OBB has many biological and pharmacological activities, such as anti-inflammatory, anti-colitis, and hypoglycemic effects^5,6^. Moreover, OBB exerted a pronounced therapeutic effect on non-alcoholic fatty liver disease (NAFLD)^7^. Our previous research suggested that OBB effectively attenuated ALI challenged by LPS/D-GalN^8^. However, as a protoberberine alkaloid, the predominance of hydrophobic groups over hydrophilic ones in the OBB molecule causes its poor solubility in water. The poor water solubility results in a low fraction of the administered dose being absorbed, which further leads to poor bioavailability. There is an urgent need to find ways to improve water solubility of OBB. However, there is no publication about the new drug delivery system of OBB and its potential pharmacological effect.

**Table 1:**
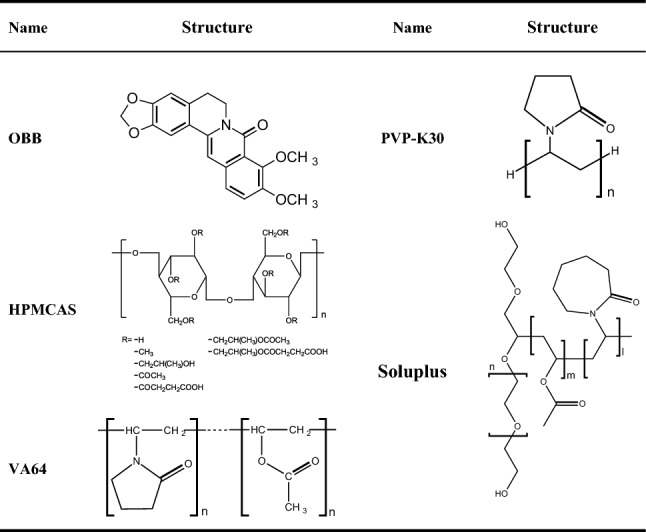
Chemical structure of the drug and excipients.

In recent years, a variety of technologies have been developed to increase the solubility of insoluble drugs in order to improve their bioavailability. These technologies include solid dispersion^9^, liposome^10,11^, micro-pulverization^12,13^, nanocrystalline^14^, eutectic^15^, etc. Among these methods, amorphous solid dispersion (ASDs) is commonly employed due to its ability to effectively enhance drug solubility and bioavailability^16^. It also offers a relatively simple and cost-effective approach that is easily self-administered^16^. Amorphous solid dispersions (ASDs) exhibit a disordered structure and possess a high free energy state, contributing to their ability to enhance dissolution rates^17^. As a sophisticated pharmaceutical system used to formulate amorphous solid dispersions, spray drying is an efficient, energy-intensive, one-step continuous, and scalable drying process. Beyond powder properties control, spray drying also possesses some key advantages like (1) shorter process cycle time, (2) high yield, and (3) low solvent residue^18^.

ASDs present a versatile solution for enhancing the solubility of challenging drug compounds. By dispersing drug molecules within a stable amorphous matrix, ASDs significantly improve drug solubility, leading to enhanced dissolution rates and increased bioavailability. Unlike techniques like nanocrystals, ASDs offer simplified manufacturing processes, making them cost-effective and scalable for large-scale production^16^. Additionally, ASDs provide flexibility in formulation design, allowing for tailored optimization of drug loading, stability, and release characteristics compared to techniques like eutectics^19^. With their wide applicability across various drug candidates, including hydrophobic compounds, ASDs emerge as a preferred option for solubility enhancement in different therapeutic classes. Moreover, ASDs enhance drug stability, guarding against degradation and crystallization, thus extending the shelf-life of pharmaceutical formulations. Compared to liposomes, which may face limitations in drug loading capacity and stability, ASDs offer a more robust solubility enhancement approach, making them an attractive choice for diverse applications in pharmaceutical development^20^. However, the tendency of the amorphous form to recrystallize on storage limits its use. To overcome the instability of amorphous drugs caused by the high free energy, certain polymers are utilized as the anti-plasticizer and/or the stabilizer to maintain the amorphous structure of the drug molecules during storage^21^.

In the present study, amorphous spray-dried powders of OBB (OBB-ASDs) were formulated. A design of experiment (DoE) approach was utilized to understand the effect of critical process and formulation parameters involved during the precipitation method. The optimized formulation was spray dried to achieve amorphous powders. Four pharmaceutically acceptable polymers including Hypromellose Acetate Succinate (HPMCAS), Polyvinylpyrrolidone K-30 (PVP-K30), Copovidone (Kollidon VA64), and polyvinyl caprolactam-polyvinyl acetate-polyethylene glycol graft copolymer (Soluplus) were applied in the spray dried formulation. The OBB-ASDs formulations were characterized by evaluating the morphology and physicochemical characterizations using SEM (scanning electron microscopy), EDS (energy dispersive spectroscopy) Mapping, X-ray diffraction (XRD), Differential scanning calorimetry (DSC), as well as Fourier transform infrared (FT-IR). Meanwhile, the pharmacokinetics of the OBB-ASDs were investigated in parallel to the OBB suspension. Furthermore, we tested the protective effect of OBB-ASDs and further explored its potential mechanism in mice with LPS/D-GalN-induced ALI.

## Materials and methods

### Materials

HPMCAS (Hydroxypropyl methylcellulose acetate succinate, H grade) was generously gifted by Ashland Global Specialty Chemicals Inc. (Covington, KY, USA). Oxyberberine (OBB, purity > 98%) was synthesized according to our previous study^6^. The chemical structure of OBB, VA64, PVP-K30, Soluplus, and HPMCAS is shown in Table 1. Soluplus, PVP-K30, and VA64 were donated by BASF (Germany). Alanine aminotransferase (ALT), aspartate transaminase (AST), malondialdehyde (MDA), superoxide dismutase (SOD), and glutathione (GSH) assay kits were obtained from Nanjing Jiancheng Bioengineering Institute (Nanjing, China). TNF-α, IL-1β, MCP-1, and IL-6 ELISA kits were purchased from Shanghai Enzyme-linked Biotechnology Co., Ltd. (Shanghai, China). Antibodies were purchased from Affinity Biosciences (OH, USA). Other reagents used in the study were of analytical grade.

### Optimization of spray drying process

**Figure 1:**
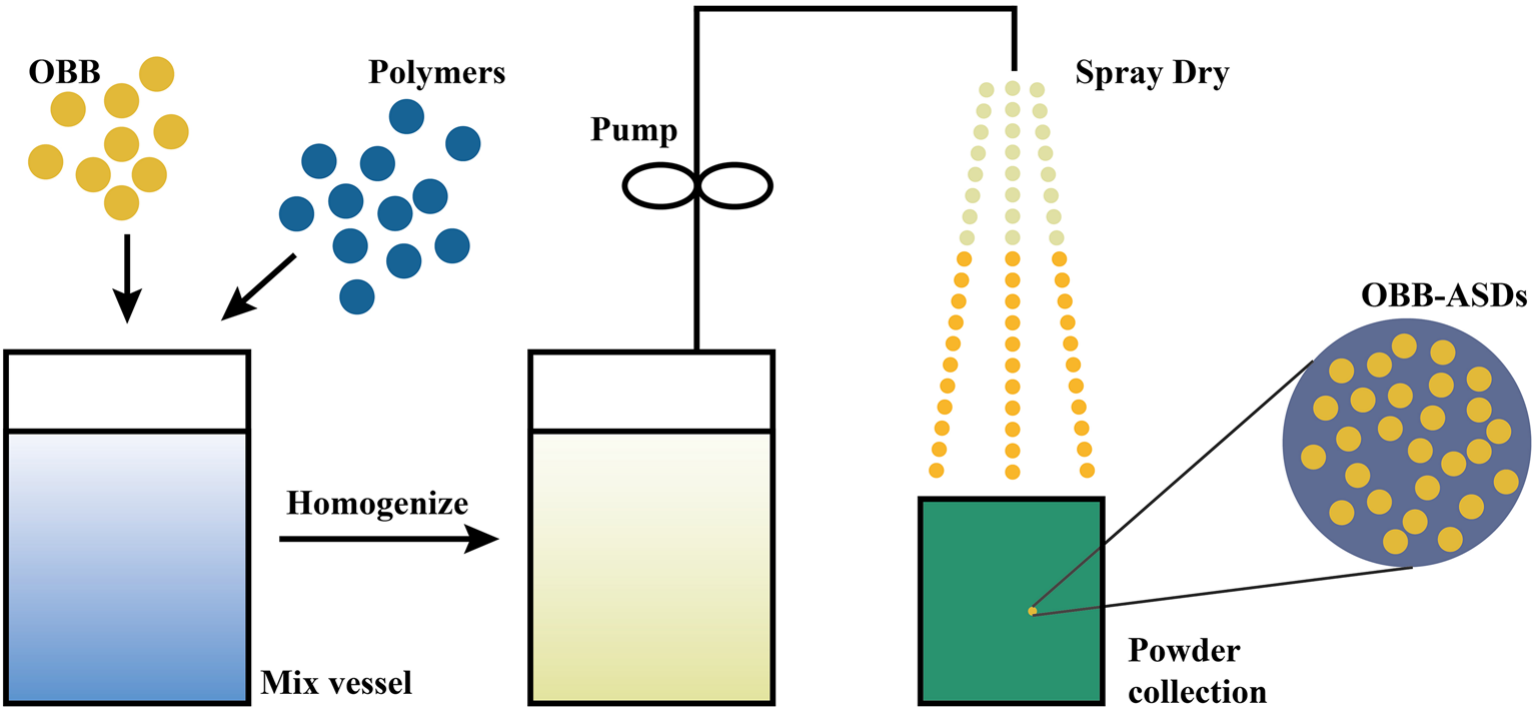
Schematic pattern of the Spray Dry.

The experimental design schematic diagram is depicted in Fig. 1. OBB dissolved in acetone to produce a different concentration. Four times OBB’s weight of HPMCAS was added to the OBB acetone solution. OBB-HPMCAS was prepared by using a spray drier BÜCHI B-290 (Flawil, Switzerland) with a standard 0.7 mm nozzle. Nitrogen was used as drying and atomizing gas in the spray dryer. Based on the pre-tests, three different critical factors drug concentration, flow rate, and temperature were chosen as independent variables. The ranges for these variables were: drug concentration 1.0–4.0 mg/mL, flow rate 1.5–4.5 mL/minute, and outlet temperature 85–115 °C. The Box–Behnken model obtained by the Design-Expert Software (Version 12, State-Ease, USA, RRID: SCR_022671) suggested 17 experiments (1 block and 5 center points per block, Table 2). The powders were collected and stored in airtight glass containers for further analysis. The product yield (%) was calculated according to the following equation:

1 $$\text{Product yield }(\%)=\frac{{W}_{n}}{{W}_{t}}\times \text{100\%}$$

where W_n_ and W_t_ are the mass of spray-dried powder, and the total mass of solid raw materials.

**Table 2:** Box–Behnken design.

Run no	Temp (°C)	Flow (mL/min)	Con (mg/mL)
1	85	3	1
2	115	3	4
3	100	3	2.5
4	100	1.5	4
5	100	4.5	1
6	115	1.5	2.5
7	100	1.5	1
8	85	4.5	2.5
9	100	3	2.5
10	100	3	2.5
11	115	4.5	2.5
12	115	3	1
13	85	3	4
14	100	3	2.5
15	85	1.5	2.5
16	100	3	2.5
17	100	4.5	4

To assess drug loading (DL), 30 mg of spray-dried samples were dissolved in 30 mL of methanol. After dissolving, solutions were filtrated and analyzed by the HPLC method^22^, and drug loading (DL) was calculated as the following equation:

2 $$\text{DL }(\%)=\frac{{Q}_{m}}{{W}_{m}}\times \text{100\%}$$

where Q_m_ and W_m_ are the weight of the detected OBB in the solution, and the weight of spray-dried samples, respectively.

### Preparation of OBB-ASDs

According to the design of experiment (DoE) results and our previous research^23,24^, we selected four polymers—HPMCAS, PVP-K30, VA64, and Soluplus—known for their excellent crystal inhibition effects to prepare the spray-drying samples. Consequently, the OBB-ASDs formulations (OBB-HPMCAS, OBB-PVP-K30, OBB-VA64, and OBB-Soluplus) were spray-dried, and the samples were characterized to further investigate their physicochemical properties. Despite the reported excellent crystallization inhibition effects of these polymers, some residual crystals were observed in certain formulations, indicating variability in their performance.

### Characterization of the OBB-ASDs

The morphology and energy dispersive spectroscopy (EDS) mapping were measured using field emission scanning electron microscope (SU8220, Hitachi, Tokyo, Japan). Powder samples was deposited onto double-sided tape and then coated with gold (thickness: 15 ~ 20 nm). Electrical potential was set as 5 kV at 10 mA for 10 min. The crystallinity of spray-dried samples was measured using an X-ray diffractometer (Model SmartLab-9k, Rigaku Corporation, Tokyo, Japan). The X-ray source used Cu target Kα-ray, the tube voltage was 40 kV, the tube current was 40 mA. All the scans were set at the step size of 0.02° and the scanning rate of 5°/minute from 5° to 45° at 2θ ranges. The results were normalized and analyzed using the Jade software (Version 6.5, MDI Instruments, USA). Samples were investigated calorimetrically using differential scanning calorimetry (DSC, Q2000, TA Instruments, USA). Powder samples of 5 mg were weighed before being hermetically sealed in aluminum pans and heated from 50 to 300 °C at a rate of 10 °C/min. Nitrogen was used as the purge gas and protective gas at flow rates of 50 mL/min and 150 mL/min, respectively. Moreover, FT-IR was applied to collect the infrared spectrum of spray-dried samples using a Nicolet iS5 FT-IR spectrometer (Thermo Scientific, USA). 5 mg powder samples and 100 mg potassium bromide were pulverized in a mortar and pressed into a disk. Spectra were collected at a range of 400 ~ 4000 cm^−1^ under ambient temperature. All curves were obtained by plotting using GraphPad Prism software (version 9.1.0, California USA, RRID: SCR_002798).

### In vitro dissolution study of OBB-ASDs

The dissolution Apparatus II (stirring paddle method) was utilized for the in vitro dissolution studies at a rotation speed of 100 ± 1 rpm in 250 mL 0.3% (w/v) sodium dodecyl sulfate (SDS) aqueous solution medium with a ZRS-8G dissolution tester (Tianjin Haiyida Technology Co., Ltd, Tianjin, China). All experiments were performed at 37 ± 0.5 °C. The OBB-ASDs samples were weighed to be equivalent to 2 mg of OBB. At predetermined time points (0, 5, 10, 20, 30, 60, and 90 min), 0.5 mL of sample solution was taken out and replaced with the same volume of fresh SDS solution (37 ± 0.5 °C). After being filtered through a 0.22 μm syringe filter, the filtered samples were collected and analyzed by HPLC.

### In vivo oral bioavailability study

Male SD rats weighing 220 ± 15 g were obtained from the Guangdong Medical Laboratory Animal Center (GDMLAC, Guangzhou, China). After fasting overnight, six rats were taken in each group, based on respective treatment at a dose of 100 mg/kg each: OBB-HPMCAS and OBB-SUS (OBB suspension). After isoflurane anesthesia, blood samples were collected from the posterior orbital venous plexus at 5, 10, 20, 30, 60, 90, 120, 180, 240, 360, 480, 720, and 1440 min, respectively. After centrifugation (12,000 rpm, 5 min), plasma was collected and stored at − 80 °C for further analysis. For deproteinization of plasma, ethyl acetate (200 μL) was added to the mixture and vortexed for 3 min. After centrifugation for 10 min (12,000 rpm, 5 min), the supernatant was collected and vacuum dried at 40 °C, − 0.8 Mpa. The residue was redissolved with 100 μL methanol/water (50:50, v/v). After centrifugation (12,000 rpm, 10 min), the supernatant was analyzed by LC–MS/MS system. Pharmacokinetic parameters were calculated based on the plasma pharmacokinetics of OBB. The peak concentration (*C*_*max*_), time of the peak concentration (*T*_*max*_), half-life (*T*_*1/2*_), and area under the profile from time 0 to 24 h (*AUC*_*0-24*_), were calculated using a DAS software (Version 2.0; Drug And Statistics, Shanghai, China, RRID: SCR_022672).

### The anti-ALI activity of OBB-ASDs

#### Animals

Male C57BL/6 mice (SPF grade, aged 6–8 weeks, weighing 25 ± 1 g,) were provided by Guangdong Medical Laboratory Animal Center (Guangzhou, China, Certificate No. SCXK 2018-0034). The animals were housed in the Experimental Animal Center of Guangzhou University of Chinese Medicine (Guangzhou, China) under the following conditions: 23 ± 2 °C temperature, 50 ± 5% relative humidity, and 12 h day/light rhythm. All mice were acclimatized for 7 days before the ALI induction and allowed ad libitum access to water and food. All experiments were carried out in compliance with the Guide for the Care and Use of Laboratory Animals (NIH, eighth edition) and followed ARRIVE recommendations (Animal research: reporting in vivo experiments) guidelines. The study was approved by the Animal Experiment Ethics Committee of Guangzhou University of Chinese Medicine (Permit ID: 20210517005).

#### ALI induction and treatment

All mice were randomly divided into six groups (n = 8): normal control group (NC); LPS/D-GalN group; OBB-ASDs25 group (25 mg/kg OBB-HPMCAS); OBB-ASDs50 group (50 mg/kg OBB-HPMCAS); OBB-SUS group (50 mg/kg OBB suspension); Silymarin group (100 mg/kg Silymarin). The drug dosage design for the ALI trial was primarily based on our previous research findings^25^ and preclinical experiments. Except for NC and LPS/D-GalN groups, all other groups of mice were respectively pretreated by gavage with the corresponding dose of OBB-ASDs, OBB-SUS, and Silymarin once daily for 7 consecutive days. Mice in NC and LPS/D-GalN group were given the same volume of saline by intragastric administration. At 2 h postdose, all mice (except the NC group) were exposed to 30 μg/kg LPS and 600 mg/kg D-GalN. Mice in NC group were received the same volume of PBS intraperitoneally. The schematics of the experiment are shown in Fig. 5A. After challenging for 6 h, blood samples were collected from the eyeball vein under 2% sodium pentobarbital anesthesia. Subsequently, the liver was immediately dissected, weighed, washed with saline, and stored at − 80 °C until further analyzed.

#### Liver pathological changes evaluation

Liver tissue pathology was evaluated by regular hematoxylin–eosin (H&E) staining. In brief, liver specimens were immediately fixed in 4% (w/v) paraformaldehyde for a day at 4 °C after harvesting. Subsequently, specimens were rinsed with distilled water and dehydrated for 10 min with ascending concentration series of ethanol. After gradient dehydration, the samples were paraffin embedded, and sectioned at 5-μm thickness, Finally, sections were stained with H&E and imaged under a BX53 light microscope (Olympus, Tokyo, Japan, RRID: SCR_022568). According to the scoring system, liver injury was evaluated by pathologists blinded to the experimental protocol. Scores were determined as Dkhil et al. previously described (Table 3)^26^.

**Table 3:** Criteria for liver histological changes.

Score	Histological changes
1–3	Cases of minimal liver damage
4–8	To the mild
9–12	To moderate
13–18	Severe cases

#### Liver function analysis

After centrifugated at 3000 g for 10 min, serum was acquired and the activities of ALT and AST were measured using commercial kits (Nanjing Jiancheng, China) following the manufacturer’s instructions.

#### Liver biochemical assessment

Fresh liver tissues were homogenized in Tris–HCl buffer (pH 7.4) and centrifuged at 4500 rpm for 15 min (4 °C). Subsequently, the supernatants were collected and assayed for MDA, SOD, GSH, MCP-1, TNF-α, IL-1β, and IL-6 using its specific commercially available assay kit following the manufacturer’s instructions.

#### Western blot analysis

Total proteins were extracted from liver tissues using ice-cold RIPA lysis buffer cell lysis buffer containing 1% protease inhibitor cocktail (Thermo Scientific, USA) and the concentrations were tested by a Bicinchoninic acid (BCA) Protein Assay Kit (Beyotime Biotechnology, China). Thirty μg of protein from each sample was separated using SDS-PAGE before being transferred to a PVDF membrane. After being blocked with 5% skimmed milk, the membranes were incubated with primary antibodies against p65 (1:1000, Cat no. AF5006, RRID: AB_2834847), TLR4 (1:1000, Cat no. AF7017, RRID: AB_2835322), MyD88 (1:1000, Cat no. AF5195, RRID: AB_2837681), Lamin B (1:10,000, Cat no. AF5161, RRID: AB_2837647), GAPDH (1:10,000, Cat no. AF7021, RRID: AB_2839421) at 4 °C overnight. The following day, membranes were incubated with the secondary antibody (1:3000, Cat no. S0001, RRID: AB_2839429) for 2 h. Finally, Bands were scanned by Western Blotting Detection System (C200, Azure Biosystems, USA) and quantified by Fiji build of ImageJ software (Version 1.53c, NIH, USA, RRID: SCR_002285). GAPDH was used as an internal control.

#### Quantitative PCR

RNAs were isolated from liver tissues with Trizol reagent and cDNAs were synthesized using a commercial kit (Vazyme Biotech, China). Quantitative PCR was performed on an Applied Biosystems 7500 Real-Time system (ABI 7500, RRID: SCR_018051). Primer sequences against mouse cDNAs used in qPCR were listed as follows: *TLR4*, 5′-gagccggaaggttattgtggtagtg-3′ (forward) and 5′-aggacaatgaagatgatgccagagc-3′ (reverse); *MyD88*, 5′-agcagaaccaggagtccgagaag-3′ (forward) and 5′-gggcagtagcagataaaggcatcg-3′ (reverse); *IL-6*, 5′-cttcttgggactgatgctggtgac-3′ (forward) and 5′-tctgttgggagtggtatcctctgtg-3′ (reverse); *IL-1β*, 5′-cactacaggctccgagatgaacaac-3′ (forward) and 5′-tgtcgttgcttggttctccttgtac-3′ (reverse); and *GAPDH*, 5′-gcacagtcaaggccgagaatgg-3′ (forward) and 5′-ggtggcagtgatggcatggac-3′ (reverse).

#### Docking calculations and molecular dynamics simulation

To explore the interaction between OBB and TLR4, molecular docking was carried out by AutoDock 4.2 (RRID: SCR_012746) and AutoDock Vina 1.1.2 (RRID: SCR_011958) program. The structure of OBB (PubChem CID: 11,066) and TLR4 (PDB ID: 3FXI) were obtained from PubChem (https://pubchem.ncbi.nlm.nih.gov) and RCSB-PDB (https://www.rcsb.org) respectively. Molecular dynamics (MD) simulation of OBB-TLR4 complexes obtained from molecular docking was performed in the GROMACS release 2023 package (RRID: SCR_014565). The specific operation was performed by following our previously reported method^22^.

### Data analysis

All the results were expressed as means ± SD (standard deviations). A Shapiro–Wilk test was used to determine the normality of the data and a Levene’s test assessed the homogeneity of variance. After being determined to be homogeneous and normally distributed, the data were analyzed using one-way analysis of variance (ANOVA) followed by Tukey’s multiple-comparison post hoc test was performed with SPSS software (version 27.0.1, IBM SPSS, USA, RRID: SCR_002865). Differences were considered statistically significant at *P* < 0.05. The bar chart was created using GraphPad Prism software (version 9.1.0, California USA, RRID: SCR_002798).

### Ethics approval

For detailed information on relevant ethical standards and criteria, please refer to the sections on “The anti-ALI activity of OBB-ASDs”.

### Consent to publish

All the authors have approved the publication of the study.

## Results

### Respond surface methodology (RSM)

**Figure 2:**
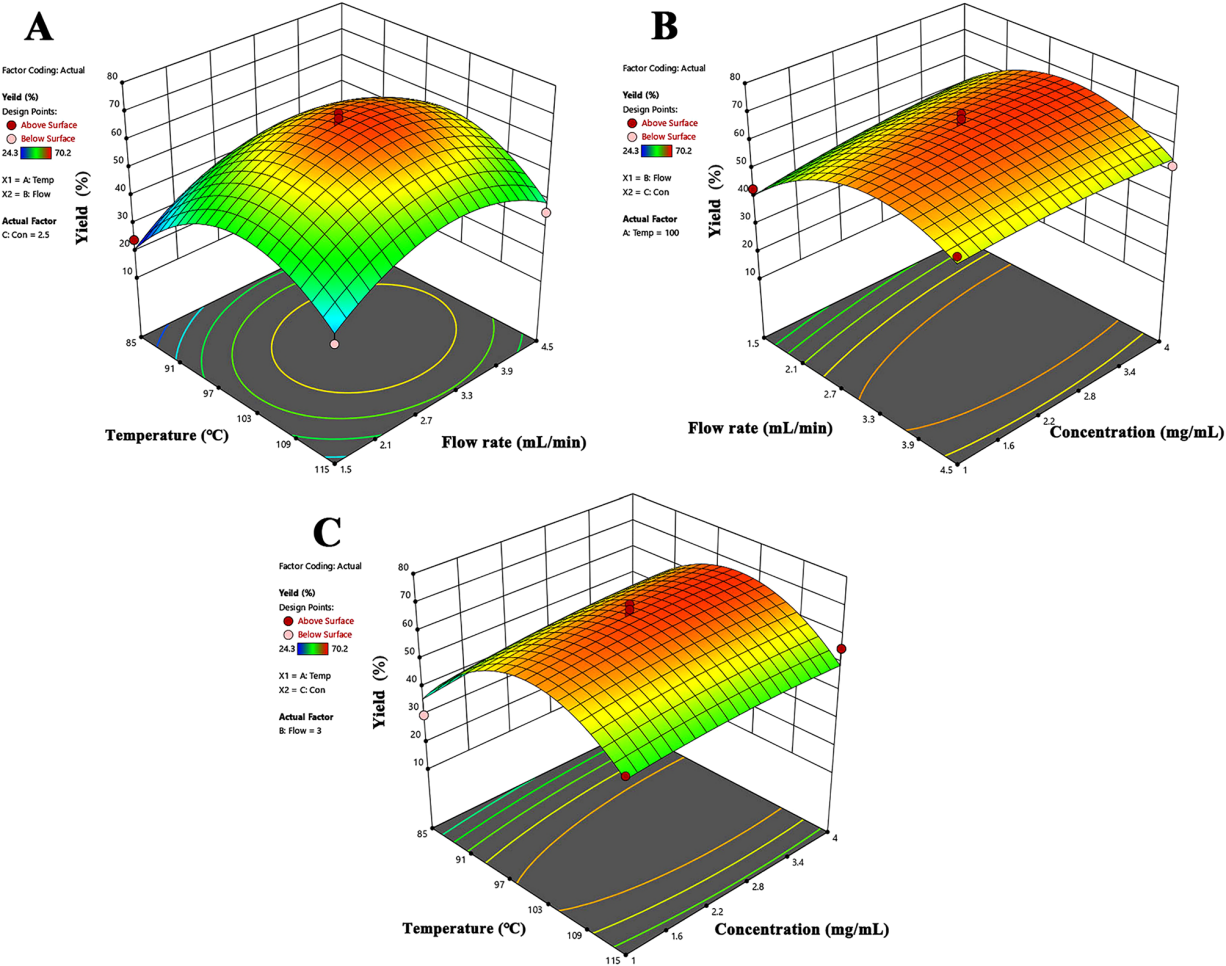
The 3D response surface plots for analysis of (**A**) Flow rate and temperature, (**B**) Flow rate and OBB concentration, (**C**) Temperature and OBB concentration on yield.

A cubic-centered design was utilized to study the factors on yield**.** 3D surface plots show the effect of flow rate, temperature, and OBB concentration on yield (Fig. 2). According to the results of RSM (Table 4), an optimization of temperature (101 °C), flow rate (3.15 mL/minute) and drug concentration (3.38 mg/mL) were chosen. Based on the ANOVA results for yield (Table 5), it is evident that the overall model is statistically significant (p-value = 0.0013), indicating that at least one of the factors (outlet temperature, flow rate, or drug concentration) significantly affects yield. Specifically, outlet temperature (A-Temp) and flow rate (B-Flow) exhibit significant individual effects on yield, with p-values of 0.0477 and 0.0344, respectively. However, interaction effects such as AB, AC, and BC, as well as quadratic terms A2 and B2, do not show significant effects on yield. The lack of fit test also reveals a non-significant lack of fit (p-value = 0.0916), suggesting that the model adequately fits the data. According to the optimization condition, drug loadings in the PVP-K30, VA64, Soluplus, and HPMCAS formulations were 16.4 ± 1.74%, 25.1 ± 4.27%, 20.3 ± 2.36% and 45.9 ± 3.31%, respectively. The product yields for the respective formulations were 43.68 ± 4.56% for PVP-K30, 31.86 ± 3.97% for VA64, 26.02 ± 2.28% for Soluplus, and 67.63 ± 3.85% for HPMCAS.

**Table 4:** Box–Behnken design results.

Run no	Temp (°C)	Flow (mL/min)	Con (mg/mL)	Yield (%)
1	85	3	1	30.2
2	115	3	4	55.4
3	100	3	2.5	70.2
4	100	1.5	4	48.8
5	100	4.5	1	58.7
6	115	1.5	2.5	29.5
7	100	1.5	1	43.2
8	85	4.5	2.5	37.5
9	100	3	2.5	66.7
10	100	3	2.5	61.4
11	115	4.5	2.5	35.7
12	115	3	1	49.3
13	85	3	4	42.5
14	100	3	2.5	68.4
15	85	1.5	2.5	24.3
16	100	3	2.5	65.7
17	100	4.5	4	52.6
18*	101	3.15	3.38	67.6

*The optimization process condition.

**Table 5:** ANOVA results for yield.

Source	Sum of squares	df	Mean square	F-value	p-value	
Model	3254.84	9	361.65	13.26	0.0013	Significant
A-Temp	156.65	1	156.64	5.74	0.0477	
B-Flow	187.21	1	187.21	6.86	0.0344	
C-Con	40.05	1	40.051	1.47	0.2649	
AB	12.25	1	12.25	0.45	0.5242	
AC	9.61	1	9.61	0.35	0.5714	
BC	34.22	1	34.22	1.25	0.2996	
A^2^	1787.21	1	1787.21	65.54	0.0001	
B^2^	840.36	1	840.36	30.82	0.0008	
C^2^	9.82	1	9.82	0.36	0.5673	
Residual	190.89	7	27.27			
Lack of fit	146.90	3	48.96	4.45	0.0916	Not significant
Pure error	43.99	4	11.00			
Cor total	3445.72	16				

### Scanning *electron* micrographs (SEM) morphology

**Figure 3:**
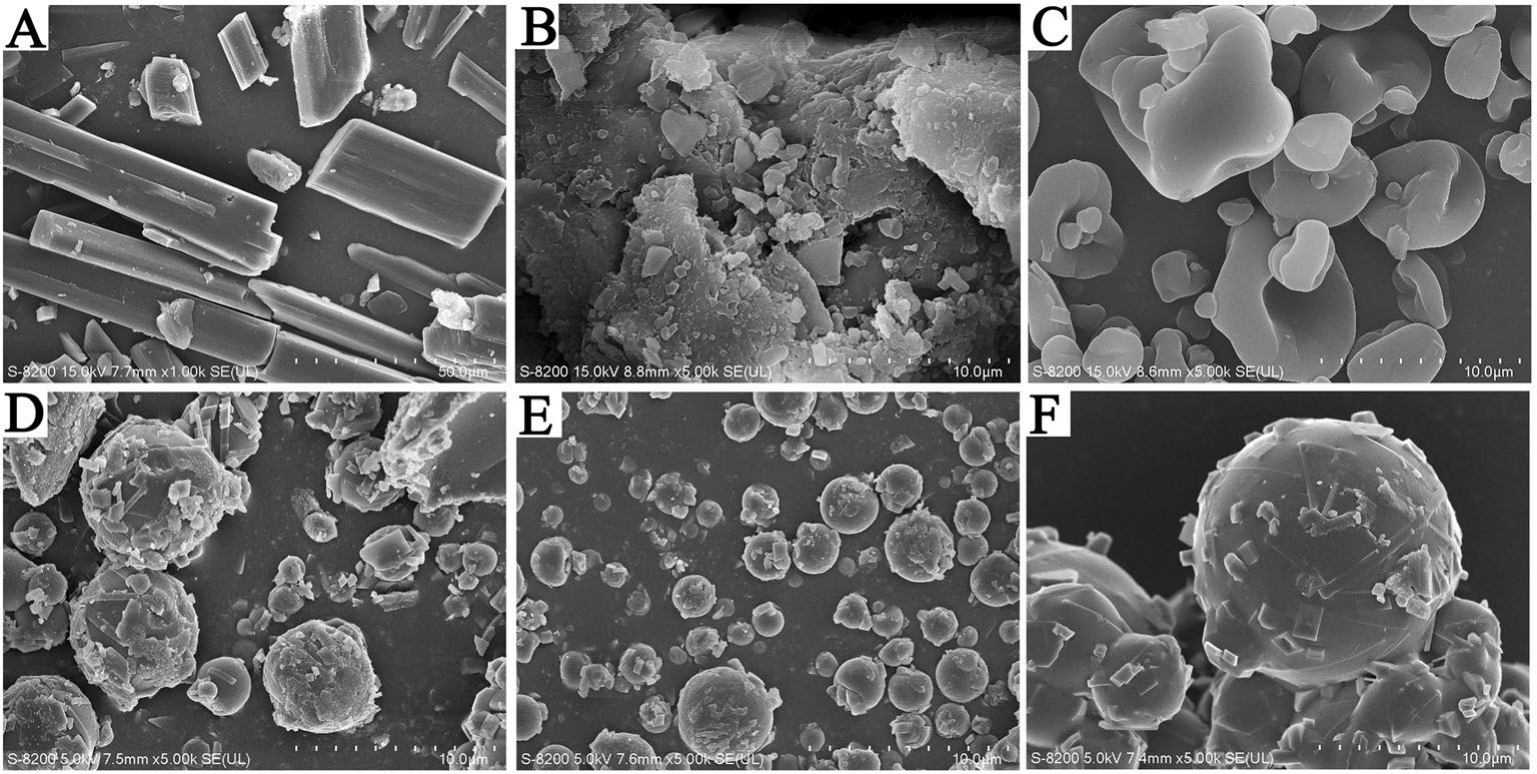
SEM micrographs. (**A**) Untreated OBB API, 1000×; (**B**) Physical blend of OBB and HPMCAS, 5000×. (**C**) OBB-HPMCAS spray-dried powder, 5000×. (**D**) OBB- Soluplus spray-dried powder, 5000×. (**E**) OBB-PVP-K30 spray-dried powder, 5000×. (**F**) OBB-VA64 spray-dried powder, 5000×.

As the original SEM (Fig. 3) shows, the morphology of OBB API appears as needle-like irregular-shaped crystals. The shapes of OBB-PVP-K30, OBB-VA64, and OBB-Soluplus were near-spherical shapes with a small amount of OBB crystal on the surface. On the other hand, spray-dried OBB-HPMCAS powder exhibited a shrink-ball shape with relatively smooth surfaces and some concavities, notable without OBB crystal appearance. The particle size of OBB-ASDs was approximately 2 ~ 10 μm, which may be attributed to the spray drying process.

### X-ray diffraction (XRD) analysis

**Figure 4:**
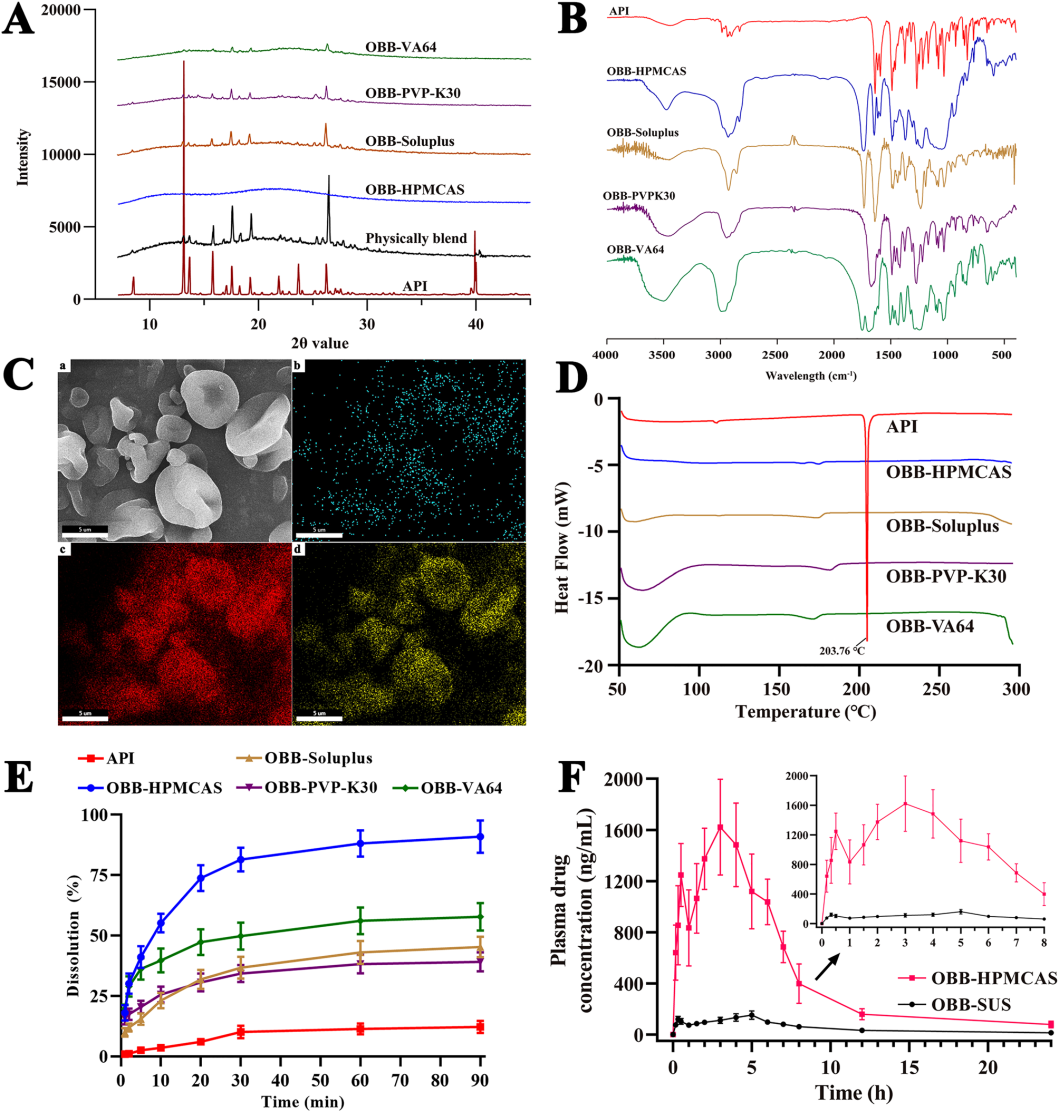
Physicochemical characterizations of processed OBB. (**A**) XRD of API and optimized spray-dried formulations; (**B**) FT-IR spectra of the API, OBB-HPMCAS, OBB-Soluplus, OBB-PVP-K30, and OBB-VA64 spray-dried powder; (**C**) EDS mapping of the composition of OBB-HPMCAS spray-dried powder. (**a**) SEM image. (**b**) N element. (**c**) C element. (**d**) O element; (**D**) DSC thermograms of the pure OBB, OBB-Soluplus, OBB-PVP-K30, and OBB-VA64 spray-dried powder; (**E**) Fig. 8 Effect of polymer type on in vitro dissolution; (**F**) Mean plasma concentration–time curves of rat after oral administration of OBB-HPMCAS and OBB-SUS.

As shown in Fig. 4A, the XRD spectrogram of OBB exhibited several intensely sharp peaks, which could be attributed to the high crystallinity of OBB. However, the number and intensity of crystallization peaks in OBB-Soluplus, OBB-PVP-K30, OBB-VA64, and OBB-HPMCAS were significantly reduced, indicating API entrapment and amorphization. Additionally, it is worth noting that the curve for OBB-HPMCAS was smooth with the OBB characteristic peaks no longer being present, suggesting the amorphous state of OBB existed in OBB-HPMCAS.

### Fourier transform infrared spectroscopy (FT-IR) analysis

The FT-IR spectra of OBB and OBB-ASDs are shown in Fig. 4B. In the OBB spectrum, the peak at 1643 cm^−1^ can be attributed to the carbonyl (C=O) bond. The other wavenumber of obvious peaks in the curve of OBB was 1594 cm^−1^ and 1619 cm^−1^ owing to the aromatic ring^27^. The spectrum of all ASDs samples showed the characteristic peaks of OBB suggesting that the spray drying process would not affect the infrared absorption characteristics of OBB, while the peak intensity was weakened due to the proportion of excipients being much larger than that of the drug.

### Energy-dispersive spectroscopy (EDS) mapping analysis

Energy-dispersive spectroscopy (EDS) elemental mapping of OBB-HPMCAS was used to confirm the distribution of C, O, and N elements in the OBB-HPMCAS. The C (red), O (yellow), and N (blue) boxes, (Fig. 4C) showed that the nitrogen element (the characteristic element of OBB) was distributed throughout the cavity structure of the particles.

### Differential scanning calorimetry (DSC) analysis

To evaluate the thermal characteristics of the API and OBB-ASDs, DSC technology was utilized. Figure 4D showed the DSC thermograms of API, OBB-Soluplus, OBB-PVP-K30, OBB-VA64, and OBB-HPMCAS. The API thermogram displays a strong and sharp melting point peak at 203.76 °C, which confirmed its crystallinity. However, thermograms for OBB-Soluplus, OBB-PVP-K30, OBB-VA64, and OBB-HPMCAS showed that the melting peak of API has almost vanished and replaced with a small shallow and broad peak which confirm they are an amorphous forms^28^. These results were consistent with the XRD data, which indicated that OBB was amorphous in the ASDs formulations.

### In vitro drug release

OBB-ASDs dissolution profiles were investigated. As shown in Fig. 4E, the free API OBB release rate was significantly slower than all formulations. In addition, OBB-HPMCAS showed 90.96% release at 90 min compared to 45.32%, 39.13%, and 57.78% release of OBB-Soluplus, OBB-PVP-K30, and OBB-VA64 at the end of the same time, respectively. Therefore, due to its excellent solubility and dissolution rate, OBB-HPMCAS was chosen to be further investigated.

### In vivo bioavailability study

Figure 4F depicts the respective concentration vs. time curve of OBB in rat plasma after oral either OBB-HPMCAS or free OBB, and the pharmacokinetic parameters are summarized in Table 6. The OBB-HPMCAS (*C*_*max*_ = 1720.49 ± 215.8 ng/ml, *T*_*max*_ = 3.08 ± 1.12 h) had a superior absorption profile compared to the free OBB (*C*_*max*_ = 168.58 ± 18.61 ng/ml, *T*_*max*_ = 4.67 ± 0.52 h). Moreover, the area under the concentration–time curves from 0 to 24 h (*AUC*_*0–24 h*_) of OBB-HPMCAS and OBB suspension was 11.34 ± 1.12 and 1.26 ± 0.13 μg/mL*h, respectively, yielding a relative bioavailability of 899%. The data above showed that the bioavailability of OBB was significantly improved after preparing as amorphous solid dispersion.

**Table 6:** Comparison of pharmacokinetic parameters between OBB-HPMCAS and OBB Suspension. (n = 6) Values are shown as the means ± SD.

Group	OBB-HPMCAS	OBB-suspension
*T*_1/2_ (h)	2.32 ± 0.29	2.99 ± 0.42
*C*_max_ (ng/mL)	1720.49 ± 215.8	168.58 ± 18.61
*AUC*_0-24_ (μg/mL*h)	11.34 ± 1.12	1.26 ± 0.13
*T*_max_ (h)	3.08 ± 0.92	4.67 ± 0.52
Relative bioavailability	8.99	1

### Assessment of hepatoprotective activity

**Figure 5:**
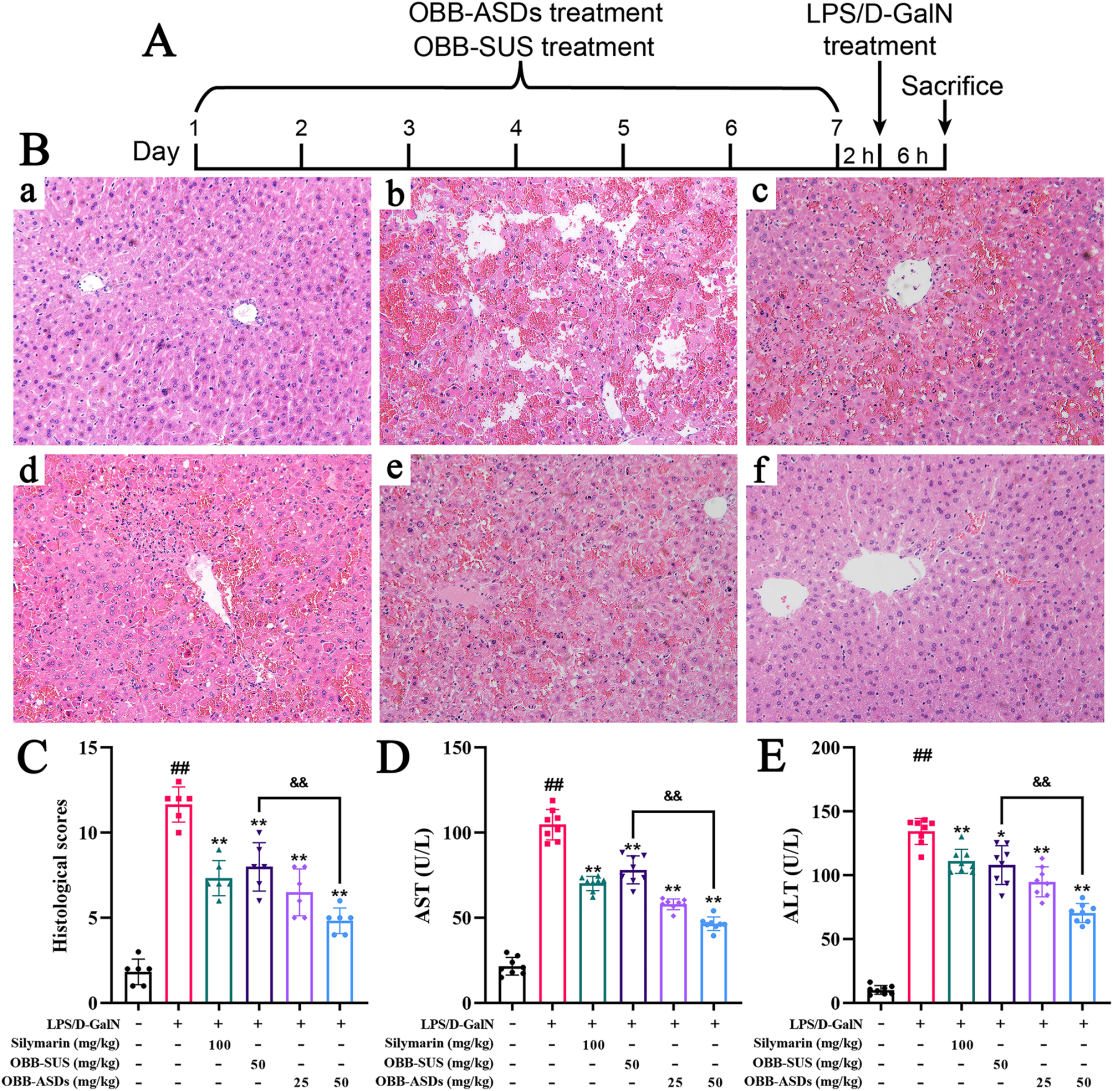
Effects of OBB-ASDs on mice experimental acute liver injury (ALI) induced by LPS/D-GalN. (**A**) Schematic of the experimental protocol, (**B**) Histopathological analysis of liver, (**C**) Histological scores, (**D**–**E**) The levels of serum transaminases including AST and ALT. (**a**) Normal control; (**b**) LPS/D-GalN; (**c**) Silymarin (100 mg/kg); (**d**) OBB (50 mg/kg); (**e**) OBB-ASDs (25 mg/kg); (**f**) OBB-ASDs (50 mg/kg). Results are means ± SD (n = 6). ^#^*P* < 0.05, ^##^*P* < 0.01 vs. Normal control group; *P < 0.05, **P < 0.01 vs. LPS/D-GalN group; ^&^*P* < 0.05, ^&&^*P* < 0.01 vs. OBB-SUS group.

As demonstrated by H&E staining (Fig. 5B–C), no significant histologic lesions were observed in either the control group. In contrast, the model group exhibited architecture disruption, inflammatory cell infiltration, and hemorrhage as compared to the control group. OBB-ASDs, OBB-SUS, and silymarin treated groups conspicuously mitigated liver tissue injury in comparison to model group. In particular, 20 mg/kg of OBB-ASDs treatment exerted a more ideal effect with a lower dose than OBB-SUS. Consistent with the pathological changes in the liver tissue, elevated ALT and AST in serum were detected in mice of LPS/D-GalN group as compared with the control group (*P* < 0.05, Fig. 5D–E). Similarly, OBB-ASDs, OBB-SUS, and silymarin pretreatment extremely reduced serum ALT and AST levels (*P* < 0.05). OBB-ASDs (20 mg/kg) displayed a superior effect protective effect than OBB suspension (*P* < 0.05). These results indicated that OBB exerted strong potency against LPS/D-GalN-induced ALI in a formulation-dependent manner.

### Effects of OBB-ASDs on oxidative and inflammatory stress

**Figure 6:**
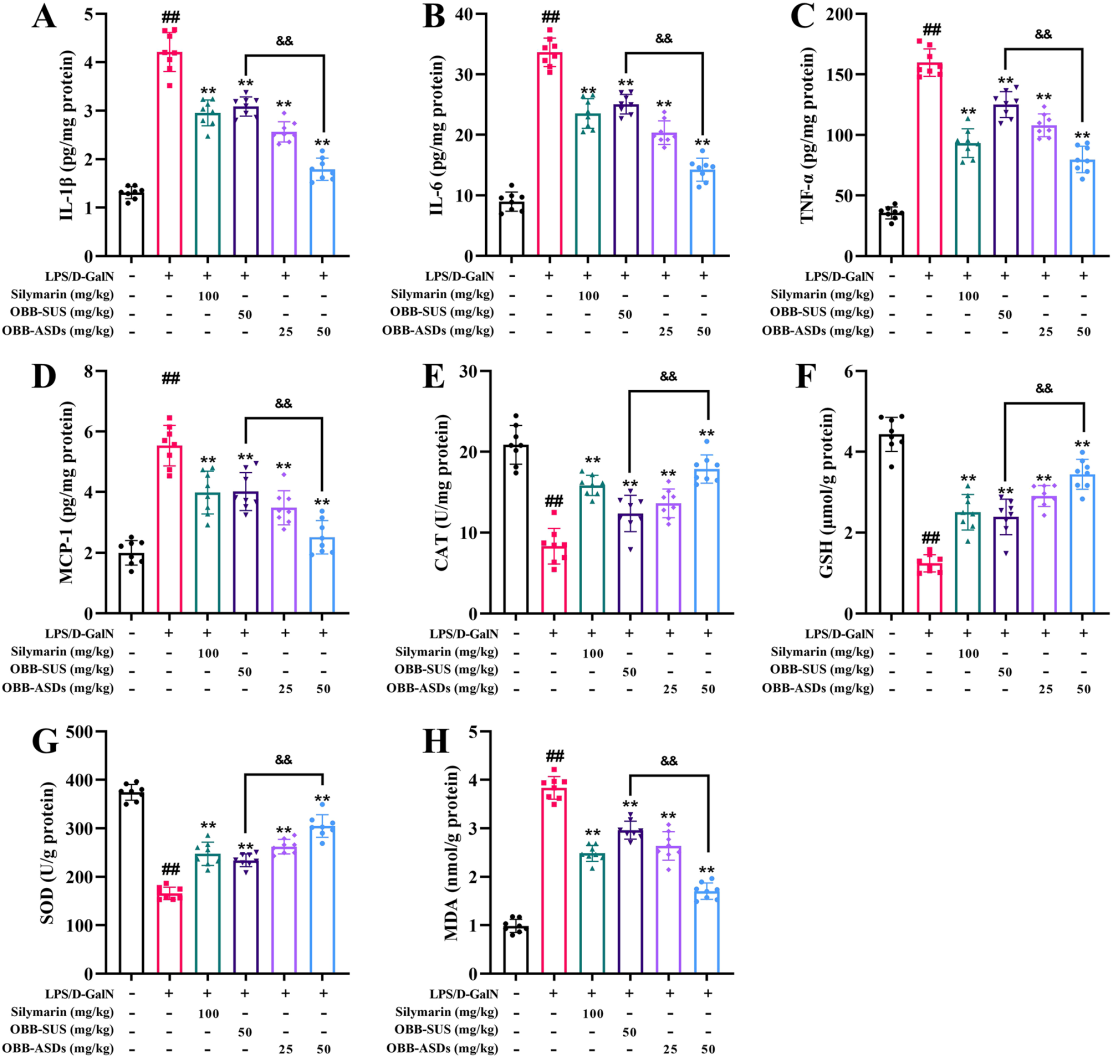
Effects of OBB-ASDs on oxidative and inflammatory stress. (**A**) IL-1β, (**B**) IL-6, (**C**) TNF-α, (**D**) MCP-1, (**E**) CAT, (**F**) GSH, (**G**) SOD, (**H**) MDA. Results are means ± SD (n = 6). ^#^*P* < 0.05, ^##^*P* < 0.01 vs. Normal control group; *P < 0.05, **P < 0.01 vs. LPS/D-GalN group; ^&^*P* < 0.05, ^&&^*P* < 0.01 vs. OBB-SUS group.

As illustrated in Fig. 6, LPS/D-GalN administration significantly increased pro-inflammatory mediators (MCP-1, TNF-α, IL-6, and IL-1β) in comparison to the healthy mice (*P* < 0.01). In contrast, OBB-ASDs, OBB-SUS, and silymarin treatment largely decreased the levels of MCP-1, TNF-α, IL-6, and IL-1β. In addition, this study also detected the mRNA expression levels of IL-6 and IL-1β by qRT-PCR. Obviously, the IL-6 and IL-1β mRNA expression levels were increased after LPS/D-GalN exposure, while OBB-SUS and OBB-ASDs could markedly restore this elevation to a large extent (*P* < 0.01). Furthermore, OBB-ASDs, OBB-SUS, and silymarin treatment also dramatically suppressed MDA levels and relieved oxidative stress by increasing hepatic GSH, CAT, and SOD contents compared with the LPS/D-GalN group. Furthermore, as compared with OBB suspension, OBB-ASDs possessed stronger antioxidant and anti-inflammatory activities.

### Effects of OBB-ASDs on NF-κBp65, TLR4, and MyD88 protein expression in liver tissues

**Figure 7:**
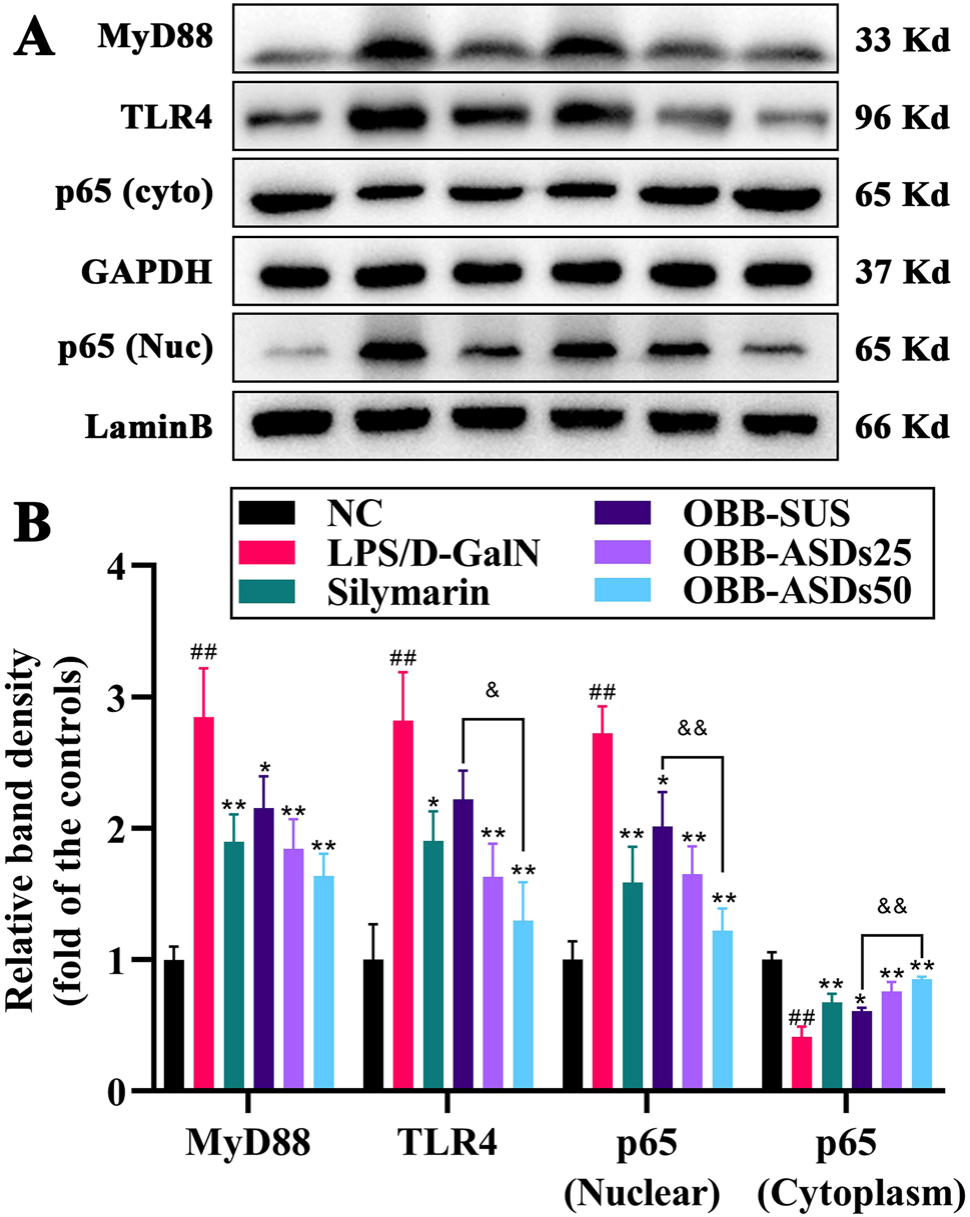
Effects of OBB-ASDs on TLR4/NFκB signaling pathway in LPS/D-GalN induced ALI mice. (**A**) Representative bands, (**B**) Quantitative results. Results are means ± SD (n = 3). ^#^*P* < 0.05, ^##^*P* < 0.01 vs. Normal control group; *P < 0.05, **P < 0.01 vs. LPS/D-GalN group; ^&^*P* < 0.05, ^&&^*P* < 0.01 vs. OBB-SUS group.

**Figure 8:**
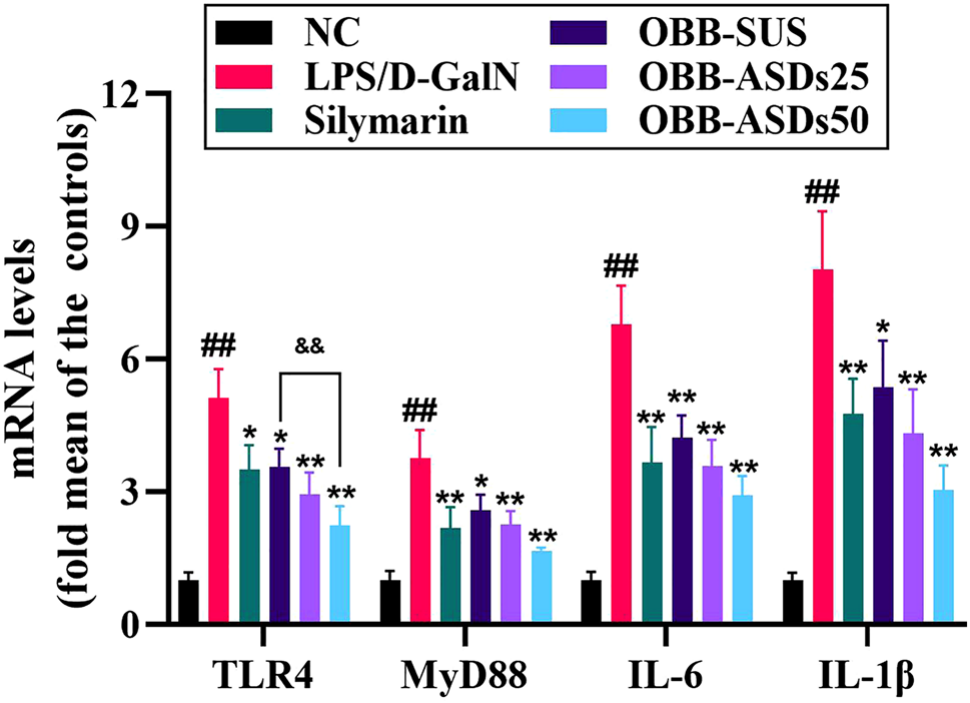
The mRNA expression levels of TLR4, MyD88, IL-1β, and IL-6. Results are means ± SD (n = 3). ^#^*P* < 0.05, ^##^*P* < 0.01 vs. Normal control group; *P < 0.05, **P < 0.01 vs. LPS/D-GalN group; ^&^*P* < 0.05, ^&&^*P* < 0.01 vs. OBB-SUS group.

The protein expressions of NF-κBp65, TLR4, and MyD88 were distinctly elevated in the LPS/D-GalN-treated mice when compared with normal control mice (*P* < 0.05, Fig. 7). However, OBB-ASDs treatment effectively suppressed the expressions of NF-κB p65, NF-κB p50, TLR4 and MyD88 in the liver tissues of LPS/D-GalN-treated mice (*P* < 0.05). Additionally, real-time PCR indicated that the expressions of TLR4 and MyD88 were higher in LPS/D-GalN challenged mice, which were restored by OBB-SUS and OBB-ASDs pretreatments (*P* < 0.01, Fig. 8).

### Molecular modeling of the binding of OBB to TLR4

**Figure 9:**
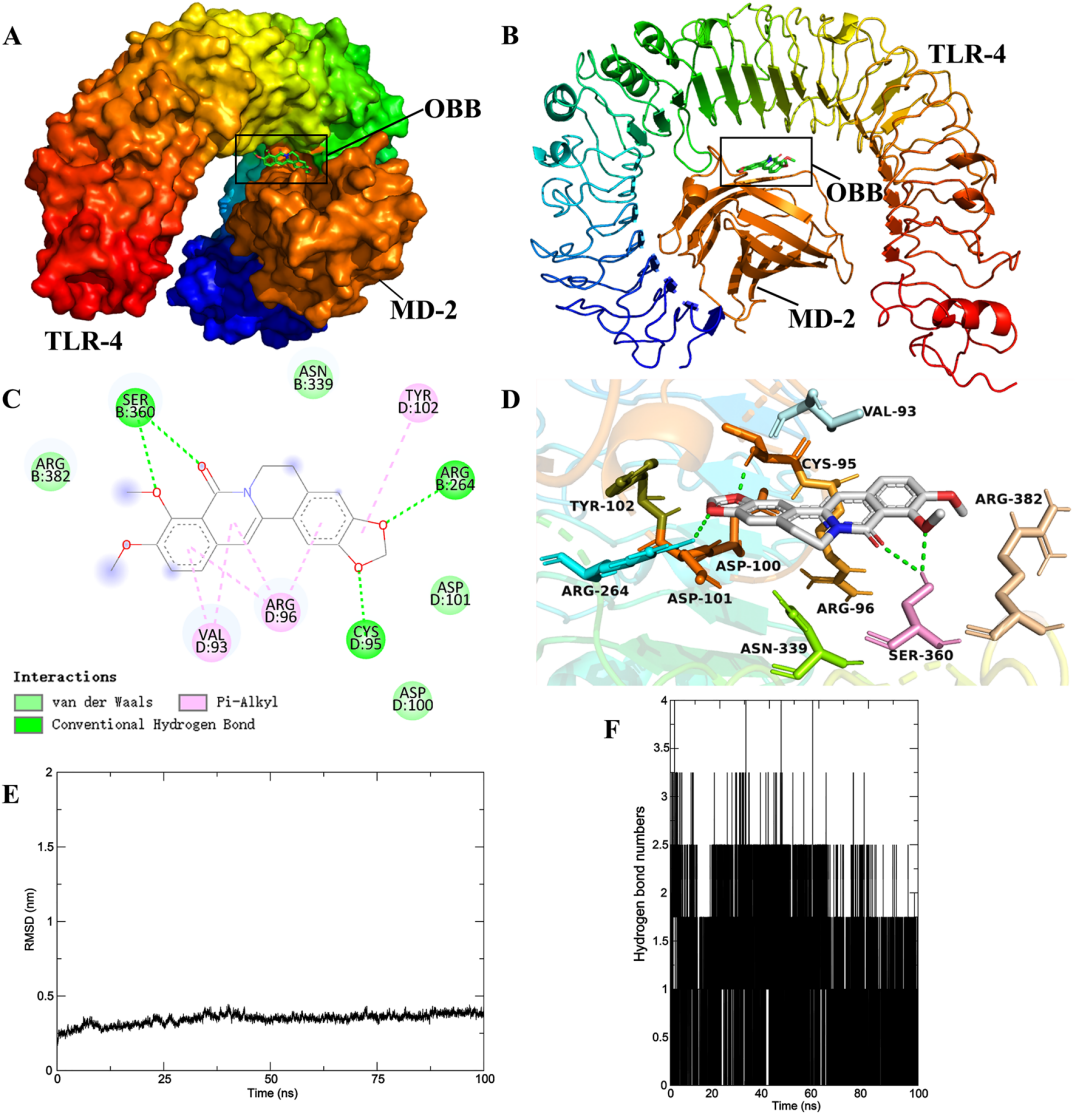
Schematic diagram showing the interaction of OBB with TLR4 in molecular docking and MD simulation analysis. (**A**,**B**) Putative binding mode of OBB with TLR4, (**C**,**D**) Docked orientation of OBB with depiction of corresponding amino acid residues of TLR-4, (**E**) RMSD curves. (**F**) The number of hydrogen bonds.

To probe whether OBB could directly bind with TLR4, we performed molecular docking of OBB in a 3D model of the TLR4. Docking calculations point out that OBB is a suitable ligand for the X-ray crystallographic structure of TLR4 (Fig. 9A–D). The binding energy between OBB and TLR4 is − 7.2 kcal/mol (Table 7). In addition, OBB could form four hydrogen bonds with the binding pocket of TLR4 (SER360, CYS95, and ARG264). The best conformation obtained from docking was used for 100 ns MD simulation analysis. The RMSD value of the TLR4 backbone was stable during the 100 ns MD trajectory (Fig. 9E). Furthermore, a maximum of four hydrogen bonds were formed during the simulation, which was congruent with the molecular docking results (Fig. 9F).

**Table 7:** Docking score of OBB with TLR4.

Protein molecule	Chemical compound	Docking score				
		Binding energy (kcal/mol)	H-bond	Amino acid	Ligand atom	H-bond length (Å)
TLR4 (3FXI)	OBB CID: 11066	− 7.2	4	SER360	O23	2.34
				SER360	O3	2.95
				CYS95	O1	2.57
				ARG264	O2	1.84

## Discussion

OBB, a novel oxoprotoberberine alkaloid exhibits numerous pharmacological effects^5^. Our previous studies suggested that OBB exerted a significant hepatoprotective effect against ALI and NAFLD^7,8^. However, OBB is a typical fat-soluble compound that can be dissolved in nonpolar solvents, while poorly soluble in water^29^. Furthermore, our preliminary experiment showed that pure OBB had poor absorption and oral bioavailability, which might hamper its transition from research to clinical application. Therefore, in an effort to improve the solubility and oral bioavailability of OBB, the development of new drug delivery systems are in urgent demand.

Carrier-mediated transportation, as well as passive diffusion, are the two main ways of drug absorption, in the gastrointestinal (GI) tract. However, the amount of free drug in aqueous solution determines the extent of drug absorption in the GI tract, regardless of which routes^30^.

Constructing an ASDs delivery system, in which API was freely dispersed in the hydrophilic polymer, is an ingenious strategy to improve solubility. It is well known that a supersaturated drug solution, which is generated by amorphous formulations dissolution, can overcome the solubility limited in intestinal absorption^31^. In recent years, numerous studies have reported that ASDs can enhance the in vitro performance as well as in vivo bioavailability of API in animals^32,33^.

Although the amorphous form of a pharmaceutical can dramatically improve solubility and bioavailability compared to crystalline forms, the amorphous state is metastable and high energy^34^. It has been proposed that polymers can restrict crystallization by decreasing the molecular mobility of API in the amorphous state. The polymer can form strong intermolecular interactions with API and raise the glass transition temperature of dispersion due to the decrease in mobility^35^. Therefore, to prevent aggregation and crystallization, additives are necessary for pharmaceutical formulations.

In this study, spray drying was applied to formulate the amorphous powder of OBB. To delay the crystallization of OBB, four different polymers (Soluplus, PVP-K30, VA64, and HPMCAS) were applied in the formulations. As a mixture of acetic and monosuccinic acid esters of HPMC, HPMCAS is reported as an excellent precipitation inhibitor of several insoluble drugs^36,37^. PVP-K30, a hydrosoluble carrier made from the monomer N-vinylpyrrolidone is widely applied in solid dispersions^38^. VA64 is another pharmaceutically acceptable polymer, which can slow drug molecules' mobility and inhibit nucleation and crystal growth^39^. Soluplus is an amphiphilic polyvinyl caprolactam-polyvinyl acetate-polyethylene glycol graft copolymer, which has been usually used as an ASDs carrier for a variety of Chinese medicine ingredients^40^. In the current study, XRD results showed that the characteristic crystalline peaks of OBB were significantly reduced in OBB-Soluplus, OBB-PVP-K30, OBB-VA64, and OBB-HPMCAS formulations. However, some small crystalline peaks were still present in OBB-Soluplus, OBB-PVP-K30, and OBB-VA64 formulations, indicating a certain number of crystalline states OBB existed. Interestingly, the OBB characteristic peaks no longer being present in OBB-HPMCAS formulation indicating that the OBB amorphous solid dispersions were successfully prepared. Moreover, DSC data showed that OBB-HPMCAS had a weak endothermic peak, which further proved the amorphous state of OBB in the OBB-HPMCAS.

The morphology of the OBB solid dispersions is depicted in Fig. 3 (SEM), it is evident that some crystalline OBB with uneven size is distributed on the surface of OBB-PVP-K30, OBB-VA64, and OBB-Soluplus. However, SEM results showed that OBB-HPMCAS were hollow and crumpled spherical particles with a 3–10 μm particle size and no obvious crystalline OBB on the surface, which was consistent with the XRD results. Additionally, EDS mapping and element line scanning of OBB-HPMCAS indicated the characteristic N element (belonging to OBB) was homogeneously distributed throughout the cavity structure of the particles. The SEM and EDS mapping analysis suggested an amorphous state of OBB evenly dispersed in the OBB-HPMCAS with a smaller particle size. Although we selected polymers with excellent reported crystallization inhibition effects, our results revealed the presence of residual crystals in some formulations. This indicates that the inhibition effects were not uniformly effective across all polymers. Future studies should focus on comparing completely amorphous solid dispersions to better evaluate the crystallization inhibition capabilities of different polymers. Addressing this aspect will provide more robust data and improve the development of effective formulations.

The release profiles showed that all the formulations led to higher dissolution rates as compared with the pure OBB. There's a widely-held belief that due to forced solubilization in the hydrophilic carriers, drug was in its supersaturated state, which can improve wettability and dispersibility^41^. Significantly, among those four formulations, HPMCAS showed a great dissolution rate. This result coincides closely with many previous reports, which showed that HPMCAS was more effective than PVP in restraining crystal growth^42^. As we all know, the polymer is the key factor related to the drug dissolution rate from solid dispersions^43^. Due to a high succinyl substituent ratio, HPMCAS has high hydrophilicity, which correlates with high dissolution rates for API^44^. The surface of poorly water-soluble drugs interacts with HPMCAS molecules, resulting in the suppression of molecular mobility and contributing to the inhibition of crystal growth.

Due to its excellent solubility and dissolution rate, we further evaluated the in vivo bioavailability of OBB-HPMCAS. It is well known that the key influence factor in oral absorption is the free concentration of an amorphous drug. Maintaining a supersaturated state by the addition of appropriate polymers increased amorphous drug absorption due to increased drug concentration^45^. Amorphous aggregates can offer a high and sustained flux through the membrane in a high surface area and pre-wetted state. Furthermore, a high concentration of amorphous provides extra transportation mechanisms for drugs through the membrane and improves absorption^46^. Multiple studies have reported that the oral bioavailability of poorly soluble drugs could be improved by adding HPMCAS^47,48^. In the current study, the higher values of *C*_*max*_ observed in OBB-HPMCAS were consistent with the prior in vitro finding. Furthermore, a much higher *AUC*_*0-24*_ was also observed in OBB-HPMCAS in comparison to free drug suspension.

Next, the hepatoprotective effect of OBB-HPMCAS in parallel to OBB-suspension was evaluated. As D-GalN increases the sensitivity of rodents to LPS-induced hepatotoxicity, acute co-administration with LPS/D-GalN is a wide method to establish an experimental ALI model in mice^49^. In the current study, an LPS/D-GalN-induced ALI model was used to further verify the potential role of OBB-ASDs. HE staining results illustrated that marked hemorrhage, necrosis, and inflammatory cell infiltration were observed after LPS/D-GalN challenge. These pathological changes were significantly rescued by OBB-ASDs and silymarin pretreatment.

It is reported that two aminotransferases, AST and ALT were released into the circulatory system in the pathological condition of liver, due to changes in the permeability of hepatic cell membranes^50^. To assess the severity of the liver injury, serum levels of ALT and AST were determined. As expected, the serum ALT and AST levels were notably increased after co-administration with LPS/D-GalN, which were markedly reduced by OBB-ASDs pretreatment. Therefore, the beneficial therapeutic effects of OBB against ALI might be mainly depended on the improved pharmacokinetic properties of ASDs.

It is believed that inflammation response is the primary cause of ALI induced by LPS/D-GalN^51^. As key pro-inflammatory mediators, IL-6, IL-1β, and TNF-α are known to play a major role in inflammatory processes and hepatic damage^52^. Consistent with the previous research, these pro-inflammatory cytokines levels were significantly increased in LPS/D-GalN challenged mice. However, pretreatment of OBB-ASDs dramatically suppressed the levels of these cytokines. Furthermore, as the liver is a major organ attacked by ROS, oxidative stress is strongly related to the incidence of ALI^53^. Under physiological conditions, both non-enzymatic and enzymatic antioxidant defense mechanisms are involved to neutralize oxidative stress. Thus, non-enzymatic antioxidants such as GSH and antioxidant enzymes like SOD, CAT, and GSH-Px are often applied to estimate oxidative stress levels^53^. As a product of unsaturated lipid degradation by ROS, MDA is another reliable index to reflect the changes of oxidative stress and ROS production^54^. In present study, SOD and GSH levels were significantly decreased after LPS/D-GalN challenged, while MDA level was dramatically increased. However, pretreatment with OBB-ASDs markedly increased the activities of SOD, GSH, and CAT but suppressed the production of MDA. In conclusion, these observations indicated that OBB-ASDs alleviated ALI in mice by attenuating oxidative stress and the inflammatory response.

Studies have shown that TLRs, a group of receptors that are implicated in innate immunity as well as inflammation, are the main receptors for the recognition of LPS and also serves as critical site for LPS-induced liver injury^55^. When LPS recognition by TLR4 activates a series of cascades, elevates the recruitment of MYD88 and ultimately accelerates NF-κB dissociation, resulting in the release of inflammatory cytokines^56^. Therefore, TLR4/NF-κB signaling pathway emerged as an important player in the development of LPS/D-GalN-induced ALI. Data from present study illustrated that OBB-ASDs treatment inhibited the up-regulation of TLR4, MyD88, and NF-κBp65 induced by LPS/D-GalN. Furthermore, molecular docking and MD simulation results also showed a favorable molecular interaction between OBB and the TLR4 cavity, which has further confirmed the inhibitory effect of OBB on TLR4. Thus, the results of this study suggested that OBB-ASDs exerted its hepatoprotective effect in mice probably associating with the inhibition of TLR4/NF-κB pathway and further suppression of the inflammation.

However, the study primarily relied on once-daily administration of the drug, overlooking potential variations in pharmacokinetics that could affect therapeutic efficacy. Alternative dosing regimens, such as multiple daily doses or sustained-release formulations, were not explored, potentially limiting the optimization of treatment outcomes. Future research endeavors focus on exploring alternative dosing regimens that better align with the OBB's pharmacokinetic profile and clinical needs. This may involve investigating the feasibility and efficacy of more frequent dosing schedules or other administration routes to optimize therapeutic outcomes and minimize the risk of adverse events. Additionally, exploring the therapeutic effect of OBB-ASDs post-injury onset could provide valuable insights into its efficacy in managing acute liver injury and guide clinical treatment strategies.

It's worth noting that our study provides valuable insights into the prophylactic effects of OBB-ASDs, it is important to acknowledge several limitations related to the dosing regimen employed in our animal experiments. Firstly, the prophylactic dosing regimen used in this study was derived from previous preclinical studies that demonstrated its efficacy in specific contexts^57–59^. However, this regimen may not fully replicate the clinical conditions under which such prophylactic treatments would be administered to human patients. For instance, the timing, dosage, and frequency of administration in a clinical setting could vary significantly based on patient-specific factors and disease progression. Secondly, our study focused on the preventive effects of OBB-ASDs in an animal model, which may not directly translate to human physiology. While animal models provide a controlled environment to study drug mechanisms and effects, there are inherent differences between animal and human biology that could impact the drug's efficacy and safety profile. Thirdly, the prophylactic approach taken in this study involved administering the drug prior to the onset of disease symptoms, which may not always be feasible or practical in a clinical context where patients typically seek treatment after symptom manifestation. This discrepancy highlights the need for further studies to evaluate the drug's effectiveness when administered at different stages of disease progression.

To address these limitations, future research should consider varying the dosing regimen to more closely mimic clinical scenarios, including post-symptomatic treatment. Additionally, clinical trials are necessary to validate the translational potential of our findings and to determine the optimal dosing strategy for human patients. By explicitly noting these constraints, we aim to prevent any misapplication of our prophylactic dosing regimen by future researchers. Our findings should be interpreted within the context of these limitations, and we encourage ongoing investigation to refine and optimize prophylactic strategies for better clinical outcomes.

## Conclusion

A novel amorphous powder formulation of OBB was successfully prepared by spray drying technique and characterized by SEM, XRD, DSC, FTIR, and in vitro release studies. The formulation OBB-HPMCAS was optimized with lower crystallinity and enhanced the performance of OBB in terms of solubility and release. In particular, OBB-HPMCAS improved the oral bioavailability of OBB because of the change in crystallinity. The in vivo evaluation of the OBB-SD indicated improved hepatoprotective potential against LPS/D-GalN-induced ALI in mice. OBB-HPMCAS showed significant improvements in liver function parameters and inflammatory markers compared to pure OBB. In conclusion, amorphous solid dispersions could be considered as a promising carrier system for the oral delivery of OBB, providing enhanced hepatoprotective activity.

### Supplementary Information

Supplementary Information.

## Data Availability

The original contributions presented in the study are included in the article. Further inquiries can be directed to the corresponding authors.

## Abbreviations

ALI: Acute liver injury
ALT: Alanine transaminase
API: Active pharmaceutical ingredient
AST: Aspartate transaminase
ASDs: Amorphous spray dried powders
BBR: Berberine
CAT: Catalase
DAB: 3,3′-Diaminobenzidine
D-GalN: D-galactosamine
ELISA: Enzyme-linked immunosorbent assay
GSH: Glutathione
GCLC: Glutamate-cysteine ligase catalytic subunit
GCLM: Glutamate-cysteine ligase modifier subunit
H&E: Hematoxylin and eosin
HO-1: Heme Oxygenase-1
IL-1β: Interleukin-1β
IL-6: Interleukin-6
Keap1: Kelch-like ECH-associated protein 1
LPS: Lipopolysaccharide
MDA: Malondialdehyde
MCP-1: Monocyte chemoattractant protein -1
NQO1: NADPH quinone oxidoreductase 1
OBB: Oxyberberine
PC: Phellodendron chinense Cortex
PBS: Phosphate-buffered saline
SOD: Superoxide dismutase
STZ: Streptozotocin
TNF-α: Tumor necrosis factor-α
