# Supplementary material for: Enhancing hepatoprotective action: oxyberberine amorphous solid dispersion system targeting TLR4
Source: Sci Rep. 2024 Jun 28;14:14924. doi: 10.1038/s41598-024-65190-2 (PMC11213902; doi:10.1038/s41598-024-65190-2)

**Supplementary Information**

**Enhancing Hepatoprotective Action: Oxyberberine Amorphous Solid Dispersion System Targeting TLR4**

Tingting Chen ^a,g,1^, Qingguo Li ^b 1^, Gaoxiang Ai ^b^, Ziwei Huang ^b^, Jun Liu ^e,f^, Lingfeng Zeng ^c,d,f^, Ziren Su ^b^, Yaoxing Dou ^a,c,d,f,*^

*^a^ The Meizhou Hospital of Guangzhou University of Chinese Medicine (Meizhou Hospital of Traditional Chinese Medicine), Meizhou, China*

*^b^ School of Pharmaceutical Sciences, Guangzhou University of Chinese Medicine, Guangzhou, China*

*^c^ The Second Clinical Medical College of Guangzhou University of Chinese Medicine/Post-Doctoral Research Station, Guangzhou, China*

*^d^ The Second Affiliated Hospital of Guangzhou University of Chinese Medicine (Guangdong Provincial Hospital of Chinese Medicine), Guangzhou, China*

*^e^ Guangdong Second Traditional Chinese Medicine Hospital (Guangdong Province Enginering Technology Research Institute of Traditional Chinese Medicine), Guangzhou, China*

*^f^ Bone and Joint Research Team of Degeneration and Injury, Guangdong Provincial Academy of Chinese Medical Sciences, Guangzhou, China*

*^g^ School of Medicine, Southern University of Science and Technology, Shenzhen, China*

^1^ These authors contributed equally to this work.

* Corresponding author.

The Second Affiliated Hospital of Guangzhou University of Chinese Medicine, No.12, Jichang Road, Baiyun District, Guangzhou City, Guangdong, China (Y. Dou).

E-mail addresses: yaoxingdou@126.com (Y. Dou).

Phone: +86-13760723340 (Y. Dou)

**List of Contents**

**Figure S1** The apparent solubility results of OBB raw material and OBB-ASDs samples.

**Figure S2** Mice kidney histopathological Section (Hematoxylin and Eosin Staining), 200× magnification.

**Figure S3** Figure S3. Stability testing of OBB-ASDs samples for 3 weeks.

1. **Apparent solubility investigation**

Precisely weigh appropriate amounts of OBB active pharmaceutical ingredient, solid dispersion HPMCAS, PVP-K30, Soluplus, and VA64 (including 5 mg of API) into conical flasks. Add 50 mL of 0.02 mM phosphate buffer (pH 6.8) to each flask, and place them in a water bath at a constant temperature shaker (37 ± 0.5°C). Samples are taken at 5, 10, 20, 30 min, and 1, 2, 4, 8, and 24 hours, filtered, diluted, and analyzed using LC-MS/MS. Results are shown in Figure S1.


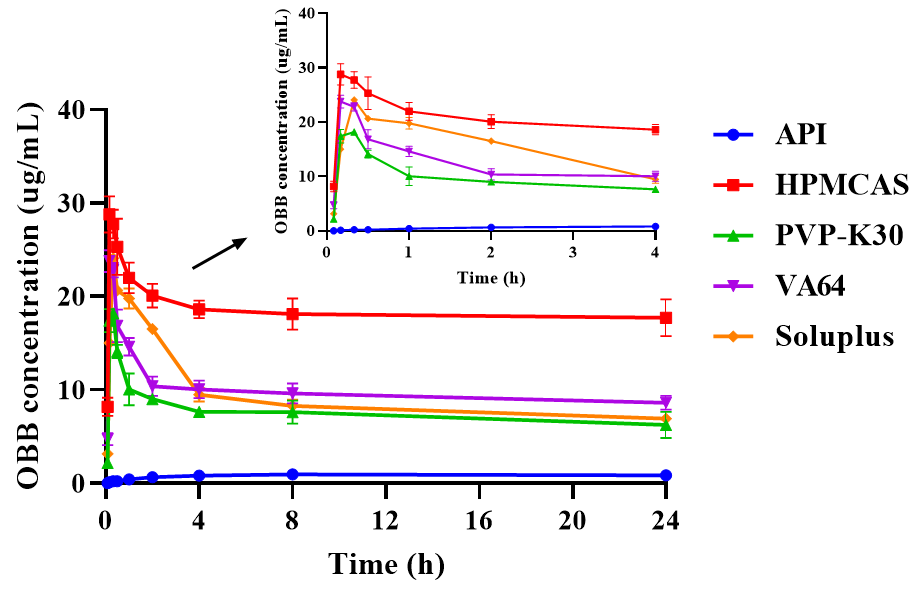


Figure S1. The apparent solubility results of OBB raw material and OBB-ASDs samples.

1. **Chronic nephrotoxicity assessment of OBB**

Male C57BL/6 mice were orally administered OBB and OBB-HPMCAS at a dose of 50 mg/kg for 16 weeks. Kidney tissues were collected at 4, 8, and 16 weeks for HE staining to observe the long-term effects of OBB administration on the kidneys. As shown in Figure S2, there was no significant kidney damage after continuous administration of OBB and OBB-HPMCAS for 4, 8, and 16 weeks.


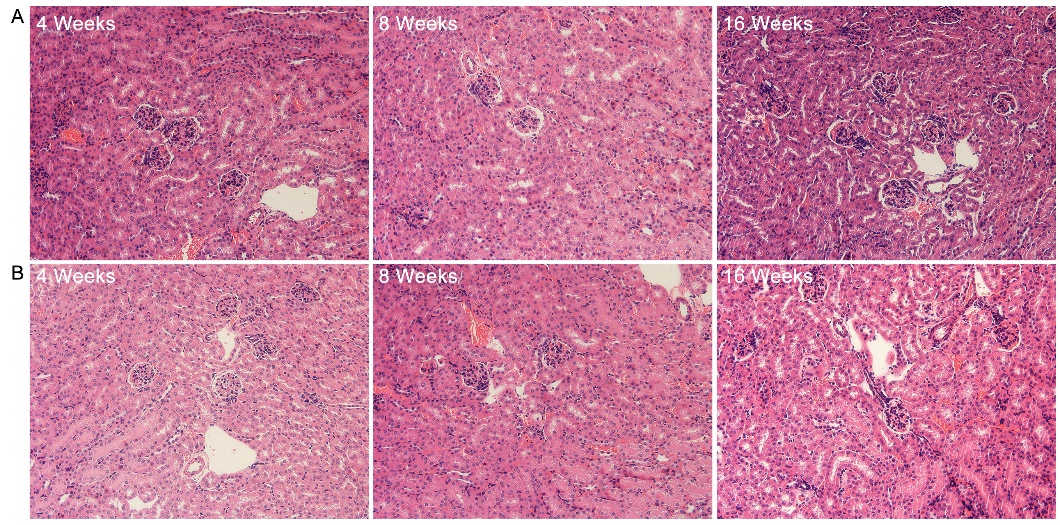


Figure S2. Mice kidney histopathological Section (Hematoxylin and Eosin Staining), 200× magnification. (A) OBB API; (B) OBB-HPMCAS.

1. **Stability testing of OBB-ASDs**

Following the sealing of the OBB-ASDs samples with aluminum foil, they were stored in a closed container under accelerated stability conditions (25±2°C, 60±5% RH) for 3 weeks. Subsequently, dissolution testing was performed on the samples to evaluate their stability. The stability results are depicted in Figure S3, indicating that the dissolution profiles of OBB-ASDs particles remained consistent after 3 weeks of storage under accelerated stability conditions.


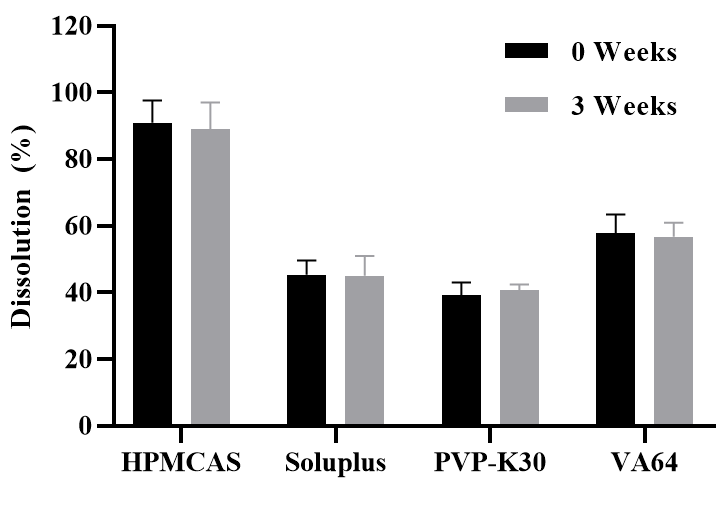


Figure S3. Stability testing of OBB-ASDs samples for 3 weeks.


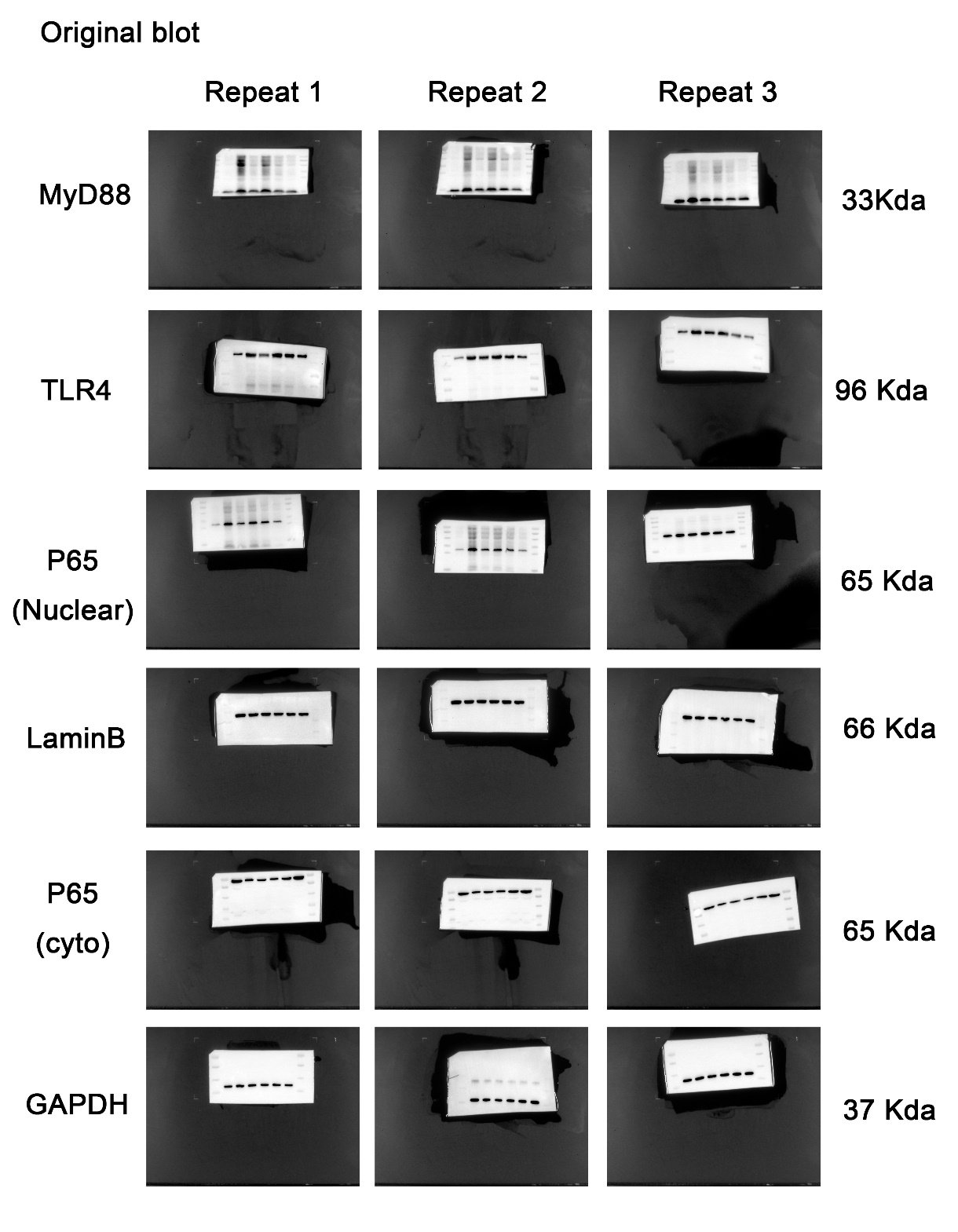

Supplement: Supplementary file 1 — Supplementary Information. [file 41598_2024_65190_MOESM1_ESM.docx]
